# Supplementary material for: Evaluation of commercially available glucagon receptor antibodies and glucagon receptor expression
Source: Commun Biol. 2022 Nov 22;5:1278. doi: 10.1038/s42003-022-04242-7 (PMC9684523; doi:10.1038/s42003-022-04242-7)
Supplement: Supplementary file 2 — Supplemental Information [file 42003_2022_4242_MOESM2_ESM.pdf]

Supplemental information

Figure S1

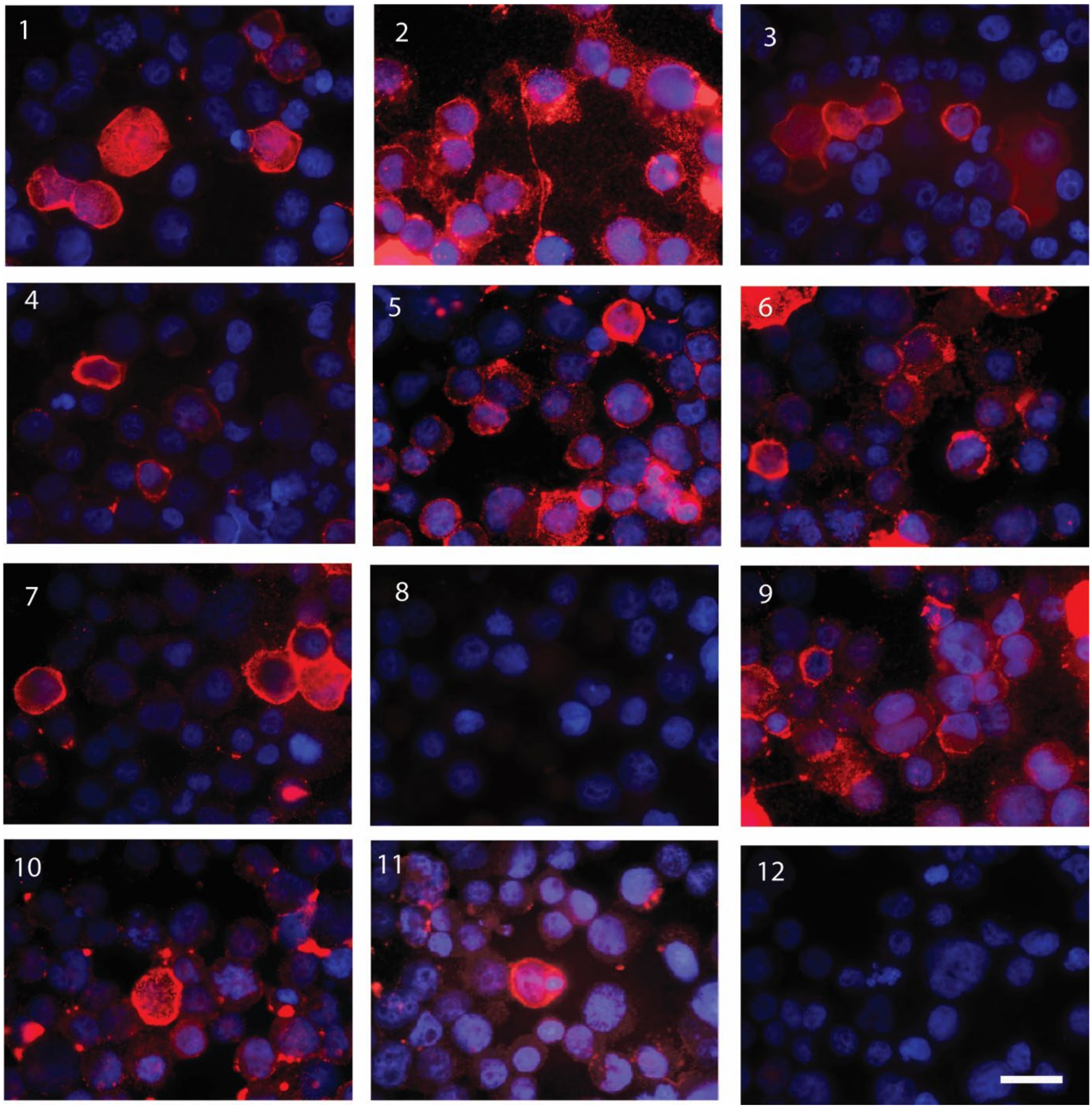

Figure S1 (Related to Figure 1). Antibody staining of transfected HEK293 cells
Extracellular GCGR antibody binding of the twelve antibodies (the number shown in each panel corresponds to the antibody listed in Table S1) showed that antibodies no. 8 and 12 did not bind extracellularly. The remaining antibodies showed varying efficiency regarding staining of the cell membrane surface of non-permeabilized HEK293 cells transiently transfected with human GCGR cDNA transcripts. Blue/purple is dapi (nuclei) and red colour is antibody binding. x390, scale bar = 30 µm. Only human GCGR vector was used: pCMV6-Entry (Cat# PS100001).

Figure S2

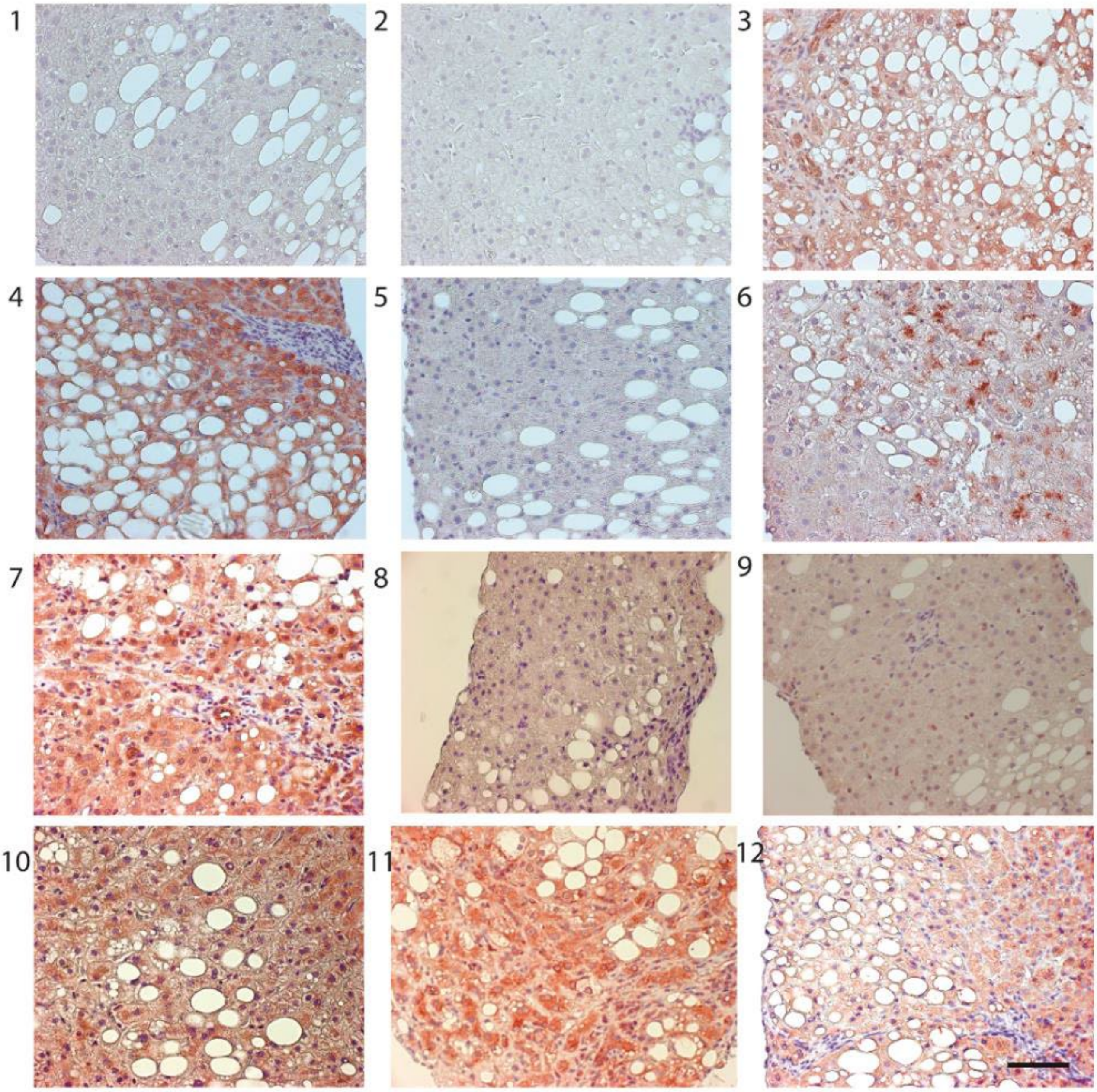

Figure S2. **Antibody staining of human liver tissue**
Paraffin-embedded liver biopsies from humans with non-alcoholic steatohepatitis (NASH) (n=3) stained using the twelve glucagon receptor (GCGR) antibodies. Antibodies no. 3, 4, 6, 7, 10, 11 and 12 showed varying staining intensity. x60, scale bar = 100 µm.

Figure S3

a MOCK transfected control with empty vector

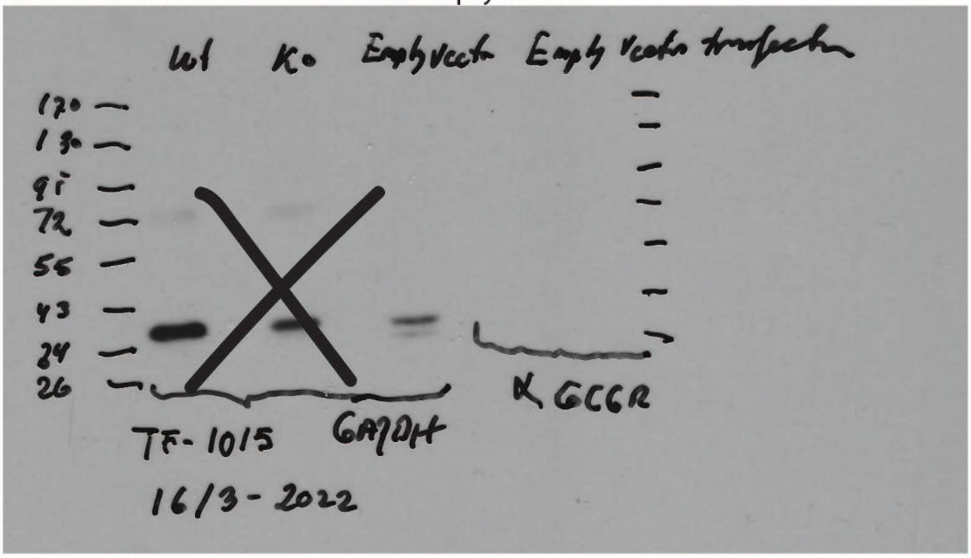

b Uncropped western blots of GAPDH loading controls of the human and mouse transfected cells

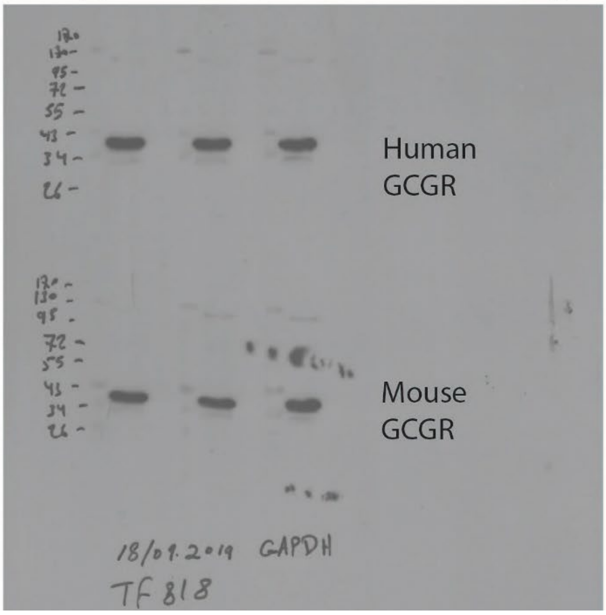

Figure S3 (related to Figure 2). Further evaluation of selected antibodies using Western Blotting
(a) Uncropped Western blotting performed with mock transfected control with empty vector. (b) Uncropped western blots of GAPDH loading controls of the human and mouse transfected cells.

Figure S4

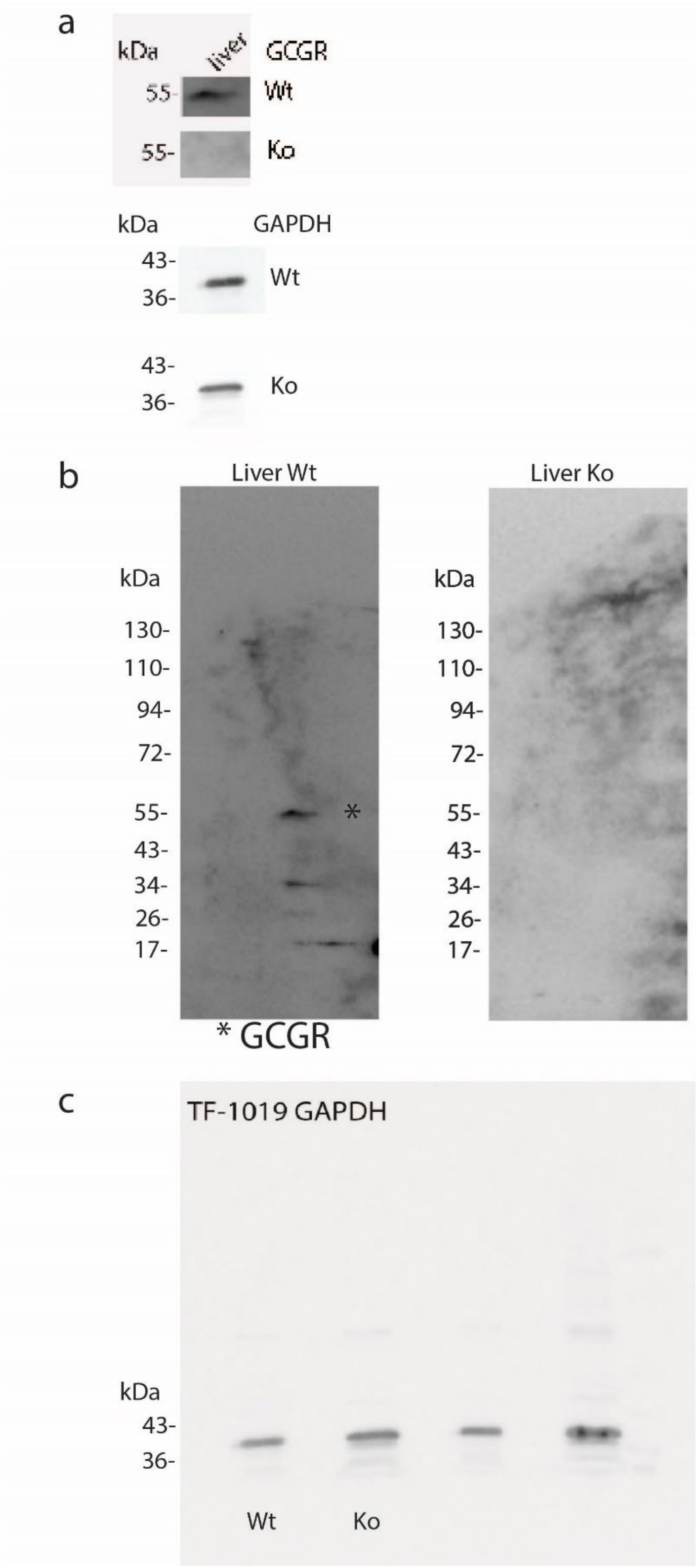

Figure S4. Further evaluation of selected antibodies using Western Blotting
(a) Cropped western blotting of lever tissue from female Gcgr$^{+/+}$ and Gcgr$^{-/-}$ mice, 8 weeks of age, using antibody no. 11 and a loading control (GAPDH). The corresponding uncropped blots are shown in panel b and c, respectively (b) Uncropped western blotting of lever tissue from *Gcgr*$^{+/+}$ and *Gcgr*$^{-/-}$ mice using antibody no. 11. The * marks the band corresponding to the predicted size of the GCGR protein. (c) Uncropped western blot of the loading control shown in panel a for the WT (*Gcgr*$^{+/+}$) and KO (*Gcgr*$^{-/-}$).

Figure S5

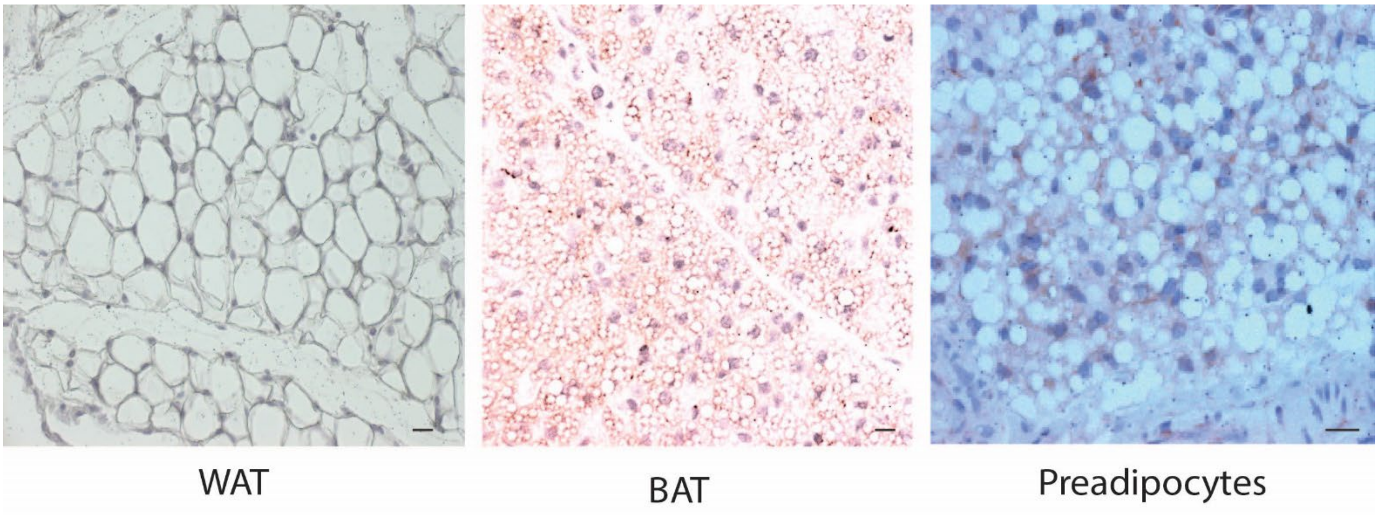

Figure S5 (Related to Figure 3). Immunohistochemical staining of adipose tissue.
Immunohistochemical staining of white adipose tissue (WAT), brown adipose tissue (BAT) and premature WAT from glucagon receptor wildtype (*Gcgr*$^{+/+}$) female mice, 8 weeks of age, using antibody no. 11. No positive immunostaining were found in WAT, whereas BAT and premature WAT showed positive staining for GCGR. WAT X100, scale bar = 30µm. BAT X75, scale bar = 40 µm. Premature WAT X80, scale bar = 60µm.

Table S1

Table S1. The twelve GCGR antibodies used in the study.
All antibodies were polyclonal and purified from rabbit immune sera. Antibody number, name of antibody, vendors, catalogue number, recommended dilution and research resource identifiers (RRID) are listed.

Antibody number	Name of antibody	Reactivity / target antigen	Vendors, Catalog #	Dilution	RRID
**1**	GCGR / Glucagon Receptor Antibody LS-C403183	Human, mouse, rat	LSbio. Cat# LS-C403183	1:40	AB_2890659
**2**	Anti-Glucagon Receptor (extracellular) antibody	Human, mouse, rat	Alomone Labs Cat# AGR-024	1:200	AB_2340975
**3**	Glucagon Receptor antibody	Human, mouse, rat	Biorbyt Cat# orb6093	1:400-800	AB_10920094
**4**	Rabbit Anti-Glucagon Receptor Polyclonal Antibody, Unconjugated	Human, mouse, rat	Bioss Cat# bs-3945R,	1:200-400	AB_10856630
**5**	Glucagon Receptor Polyclonal Antibody	Human, mouse, rat	Thermo Fisher Scientific Cat# PA5-50668	1:50-200	AB_2636120
**6**	Rabbit Anti-Human Glucagon Receptor Polyclonal Antibody, Unconjugated	Human	GeneTex Cat# GTX71693	8 µg/ml	AB_375890
**7**	Polyclonal Rabbit anti-Human GCGR / Glucagon Receptor Antibody	Human, mouse	LSbio. Cat# LS-C498919	1:100	AB_2890660
**8**	Anti-Mouse Glucagon receptor	Mouse	TriChem (cedarlane) Cat# CL8893AP	4 µg/ml	AB_2890661
**9**	Glucagon Receptor (extracellular) Polyclonal Antibody	Human, mouse, rat	Thermo Fisher Scientific Cat# PA5-77446	1:200	AB_2735850
**10**	Anti-GCGR polyclonal antibody	Human	Atlas Antibodies Cat# HPA071228	1-4 µg/ml	AB_2686368
**11**	Glucagon receptor antibody	Human	Abcam Cat# ab75240	5 µg/ml	AB_1523687
**12**	Anti-Glucagon Receptor antibody	Human	Abcam Cat# ab188743	13 µg/ml	AB_2890662

Table S2

Table S2 (Related to Figure 1). Distribution of cells GCGR/c-Myc tag positive, and only GCGR or c-Myc tag positive.
Listed are the distribution of cells that are GCGR/c-Myc tag positive, only GCGR or c-Myc tag positive when stained with the twelve antibodies. Shown for human and mouse GCGR transfected cells, respectively.

Antibody Human GCGR transfection cells	Double stained cell no.	GCGR single cell no. (red)	c-Myc tag single cell no. (Green)
No. 1	15 (100%)	0	0
No. 2	12 (100%)	0	0
No. 3	9 (100%)	0	0
No. 4	4 (57%)	3 (43%)	0
No. 5	8 (100%)	0	0
No. 6	7 (78%)	0	2 (22%)
No. 7	3 (60%)	1 (20%)	1 (20%)
No. 8	4 (26%)	11 (74%)	0
No. 9	7 (78%)	2 (22%)	0
No.10	15 (75%)	5 (25%)	0
No.11	7 (70%)	0	3 (30%)
No.12	8 (100%)	0	0

Antibody Mouse GCGR transfection cells	Double stained cell no.	GCGR single cell no. (red)	c-Myc tag single cell no. (Green)
No. 1	8 (73%)	2 (18%)	1 (9%)
No. 2	19 (100%)	0	0
No. 3	14 (88%)	0	2 (12%)
No. 4	4 (100%)	0	0
No. 5	2 (29%)	3 (42%)	2 (29%)
No. 6	7 (58%)	2 (17%)	3 (25%)
No. 7	10 (67%)	4 (26%)	1 (7%)
No. 8	7 (100%)	0	0
No. 9	2 (100%)	0	0
No.10	3 (38%)	0	5 (62%)
No.11	1 (34%)	2 (66%)	0
No.12	10 (100%)	0	0

Table S3

Table S3 (Related to Figure 1). Preparation of human and mouse GCGR transfected HEK293 cells
Sequencing of plasmids used for glucagon receptor (GCGR) expression levels investigation.

To ensure the right transcript was used, plasmids from both human and mouse were sequenced.<br>GCGR transcript from both the human and mouse plasmids was found, demonstrated below.
>H1<br>TNNNGNAATGGGCGGTAGGCGTGTACGGTGGGAGGTCTATATAAGCAGAGCTCGTTTAGT<br>GAACCGTCAGAATTTTGTAATACGACTCACTATAGGGCGGCCGGGAATTCGTCGACTGGA<br>TCCGGTACCGAGGAGATCTGCCGCCGCGATCGCCATGCCCCCCTGCCAGCCACAGCGACC<br>CCTGCTGCTGTTGCTGCTGCTGCTGGCCTGCCAGCCACAGGTCCCCTCCGCTCAGGTGAT<br>GGACTTCCTGTTTGAGAAGTGGAAGCTCTACGGTGACCAGTGTCACCACAACCTGAGCCT<br>GCTGCCCCCTCCCACGGAGCTGGTGTGCAACAGAACCTTCGACAAGTATTCCTGCTGGCC<br>GGACACCCCCGCCAATACCACGGCCAACATCTCCTGCCCCTGGTACCTGCCTTGGCACCA<br>CAAAGTGCAACACCGCTTCGTGTTCAAGAGATGCGGGCCCGACGGTCAGTGGGTGCGTGG<br>ACCCCGGGGGCAGCCTTGGCGTGATGCCTCCCAGTGCCAGATGGATGGCGAGGAGATTGA<br>GGTCCAGAAGGAGGTGGCCAAGATGTACAGCAGCTTCCAGGTGATGTACACAGTGGGCTA<br>CAGCCTGTCCCTGGGGGCCCTGCTCCTCGCCTTGGCCATCCTGGGGGGCCTCAGCAAGCT<br>GCACTGCACCCGCAATGCCATCCACGCGAATCTGTTTGCGTCCTTCGTGCTGAAAGCCAG<br>CTCCGTGCTGGTCATTGATGGGCTGCTCAGGACCCGCTACAGCCAGAAAATTGGCGACGA<br>CCTCAGTGTCAGCACCTGGCTCAGTGATGGAGCGGTGGCTGGCTGCCGTGTGGCCGCGGT<br>GTTCATGCAATATGGCATCGTGGCCAACTACTGCTGGCTGCTGGTGGAGGGCCTGTACCT<br>GCACANCCTGCTGGGCCTGGCCACCCTCCCCGAGAGGAGCTTCTTCNCCTCTACTGGGCN<br>TCGGCTGGNTGCCCCNTNCTGTNN
>H2<br>NGAGANGCCTGGGGAGGGGTCACAGGGATGCCACCCGGGATCTGTTCAGGAAACAGCTAT<br>GACCGCGGCCGGCCGTTTAAACCTTATCGTCGTCATCCTTGTAATCCAGGATATCATTTG<br>CTGCCAGATCCTCTTCTGAGATGAGTTTCTGCTCGAGCGGCCGCGTACGCGTGAAGGGGC<br>TCTCAGCCAATCTAGGGAGGCCACCAGCCAAGGGGGTCTCCGCAGATGAATCCTGGCTGC<br>CACCACCCCTCCCAAACTGCAGCTCCTTGCTGGGAGGGCCGTGGCCGGGCGAAGATGAGG<br>CCCTGTGGTTGCTGGTGTTCCGCTCCTCCCATAGCACTTTGCCCAGGCGCCAGCGGTGCC<br>AACGCCGCCGCAGCTCCGACTGCACCTCCTTGTTGAGGAAGCAGTAGAGGACAGCCACCA<br>GCAGGCCCTGGAAGGAGCTGAGGAAGAGGTCGAAGAAGAGCTTGGCGGAGCGCAGGGTGC<br>CCTGGGCGTGCTCGTCCGTCACGAAGGCGAAGACCACTTCGTGGACGCCCAGCAGAGGGA<br>TGAGGGTCAGCGTGGACTTGGCCAGCCGGAACTTGTAGTCTGTGTGGTGCATCTGCCGTG<br>CCCGCAGCTTGGCCACGAGCAGCTGAACGATGCGGACGAAGATGAAGAAGTTGATCAGGA<br>TGGCCAGGAAGACGGGGAACCGCAGGATCCACCAGAAGCCCATGTTGTCATTGCTGGTCC<br>AGCACTGGACGTTCTCGAACAGACACTTGACCACTGCCCAGGGGACGACGAACAGCATGG<br>GGGCACCCCAGCCGATGCCCAGGTAGAGGCTGAAGAAGCTCCTCTCGGGGAGGGTGGCCA<br>GGCCCAGCAGGTTGTGCAGGTACAGGCCCTCCACCAGCAGCCAGCAGTAGTTGGCCACGA<br>TGCCATATTGCATGAACACCGCGGCCNACGGCAGCCAGCCNCCGCTCNN
>M1 is M2<br>NAGGAGAGGCCTGGGGAGGGGTCACAGGGATGCCACCCGGGATCTGTTCAGGAAACAGCT<br>ATGACCGCGGCCGGCCGTTTAAACCTTATCGTCGTCATCCTTGTAATCCAGGATATCATT<br>TGCTGCCAGATCCTTCTTCTGAGATGAGTTTCTGCTCGAGCGGCCGCGTACGCGTGGTGG<br>GGCTGTCAGCCAACCTTGGGAGACTACTGGCCAGCGAGGTCTCCATAGAGGGCACACAGC<br>CAGTCCCACTGCTGCTGCCTGCACTCATAAGCTGAAGTTTCTCACAGGGATCACCATGAC<br>AAGGCCCTGCTGGGGCCATGTGGCTGCCATGGCTGCTGGCCAACCTTTCCTCCTGAAGAG<br>CTTTGCCCTTCTTGCCATTGCCTCCAACGCCGCATCAGCTCTGCCTGCACCTCCTTGTTG<br>AGGAAACAGTAGAGAACAGCCACCAGCAGACCCTGGAAGGAGCTGAGGAACAGGTCAAAA<br>AAGAGCTTGGTGGAGCGCAGGGTGCCTTGGGCATGCTCGTCAGTCACAAAGGCAAAGACC<br>ACCTCGTGGACCCCCAGCAGAGGGATGAGGGTCAGCGTGGACCTGGCCAGCCGGAACTTA

TAGTCAGCATAGTGCATCTGATGGGCACGCAGCTTGGCCACAAGAAGGTGAATGATGTGG<br>ACAAAGATGAAAAAATTGATCAGTAAGGCCAGGAAGACAGGAATACGCAGGATCCACCAG<br>AATCCCATGTTGTCATTGCTGGTCCAGCACTGAACATTCTCAAACAGACACTTGACCACC<br>ACCCCAGGGGATGACAAACAGCAGGGGCGCACCCCAGCCAATGCCCAGGTAGAGGGAAAA<br>GAAGCTCCTCTCAGAGAAGGTGGCAAGGCTCAGCAGGCTGTACAGGTACACGCCTCTANA<br>GCACCAGCATAGTTGGCTATGATGCCGTACTGCATGATCANTGTGGCNNTCTGCAGCCGG<br>CNTCGCCCGTNCTGAGC
>M2=M1<br>TNNNGNAATGGGCGGTAGGCGTGTACGGTGGGAGGTCTATATAAGCAGAGCTCGTTTAGT<br>GAACCGTCAGAATTTTGTAATACGACTCACTATAGGGCGGCCGGGAATTCGTCGACTGGA<br>TCCGGTACCGAGGAGATCTGCCGCCGCGATCGCCATGCCCCTCACCCAGCTCCACTGTCC<br>CCACCTGCTGCTGCTGCTGTTGGTGCTGTCATGTCTGCCAGAGGCACCCTCTGCCCAGGT<br>AATGGACTTTTTGTTTGAGAAGTGGAAGCTCTATAGTGACCAATGCCACCACAACCTAAG<br>CCTGCTGCCCCCACCTACTGAGCTGGTCTGTAACAGAACCTTCGACAAGTACTCCTGCTG<br>GCCTGACACCCCTCCCAACACCACTGCCAACATTTCCTGCCCCTGGTACCTACCTTGGTA<br>CCACAAAGTGCAGCACCGCCTAGTGTTCAAGAGGTGTGGGCCCGATGGGCAGTGGGTTCG<br>AGGGCCACGGGGGCAGCCGTGGCGCAACGCCTCCCAATGTCAGTTGGATGATGAAGAGAT<br>CGAGGTCCAGAAGGGGGTGGCCAAGATGTATAGCAGCCAGCAGGTGATGTACACCGTGGG<br>CTACAGTCTGTCCCTGGGGGCCTTGCTCCTTGCGCTGGTCATCCTGCTGGGCCTCAGGAA<br>GCTGCACTGCACCCGAAACTACATCCATGGGAACCTGTTTGCGTCCTTTGTGCTCAAGGC<br>TGGCTCTGTGTTGGTCATCGATTGGCTGCTGAAGACACGGTACAGCCAGAAGATTGGCGA<br>TGACCTCAGTGTGAGCGTCTGGCTCAGTGACGGGGCGATGGCCGGCTGCAGAGTGGCCAC<br>AGTGATCATGCAGTACGGCATCATAGCCAACTATTGCTGGTTGCTGGTAGAGGGCGTGTA<br>CCTGTACAGCCTGCTGAGCCTTGCCNCCTTCTCTGAGAGGAGCTTCTTTTCCTCTACTGG<br>GCATTGGCTGGGTGCGCCCTGCTGTTGNATCCCNN

Table S4

Table S4. Comparison of the specificity of the twelve antibodies
Listed are the twelve antibodies, the reactivity, positive or negative glucagon receptor (GCGR) staining on intra- and extracellular domains on transfected HEK293 cells, mouse liver tissue, and human liver tissue. In addition, staining intensity scores between 0 and 3 of liver tissues from both the glucagon receptor wildtype ($Gcgr^{+/+}$) and glucagon receptor knockout ($Gcgr^{-/-}$) mice are presented, the higher the score, the more receptor-antibody binding.

**Antibody number**	**Reactivity /target antigen, according to vendor:**	**Intra- and extracellular antibody binding of mouse GCGR transfected HEK293 cells.**	**Intra- and extracellular antibody binding of human GCGR transfected HEK293 cells.**	**Extracellular antibody binding of human GCGR transfected HEK293 cells.**	**Antibody staining of mouse liver tissue and intensity: ($Gcgr^{+/+}$/$Gcgr^{-/-}$)**	**Antibody staining of human liver tissue**
1	Human, mouse, rat GCGR	Yes	Yes	Yes	No (0/0)	No
2	Human, mouse, rat GCGR	Yes	Yes	Yes	No (0/0)	No
3	Human, mouse, rat	Yes	Yes	Yes	No (0/0)	Yes
4	Human, mouse, rat	Yes	Yes	Yes	No (0/0)	Yes
5	Human, mouse, rat	Yes	Yes	Yes	No (0/0)	No
6	Human GCGR	Yes	Yes	Yes	No (0/1)	Yes
7	Human, mouse, rat GCGR	Yes	Yes	Yes	No (0/3)	Yes
8	Mouse GCGR	Yes	Yes	No	No (0/0)	No
9	Human, mouse, rat GCGR	Yes	Yes	Yes	No (0/0)	Yes
10	Human GCGR	Yes	Yes	Yes	No (0/0)	Yes
11	Human GCGR	Yes	Yes	Yes	Yes (3/1)	Yes
12	Human GCGR	Yes	Yes	No	No (0/0)	Yes

Table S5

Table S5 (Related to Figure 5). **RNA expression of the human GCGR from various tissues and specific cells**
Number of samples per tissue used in figure 5A. Arranged alphabetically according to tissue name.

Tissue name in GTEx	Samples were taken from	Number of GTEx samples
Adipose Tissue	Subcutaneous & visceral	406
Adrenal Gland	Adrenal Gland	56
Blood Vessel	Aorta (Artery), Coronary (Artery), Tibial (Artery)	409
Brain	Amygdala, Anterior cingulate cortex (BA24), Caudate, Cerebellar Hemisphere, Cerebellum, Cortex, Frontal Cortex (BA9), Hippocampus, Hypothalamus, Nucleus Accumbens, Putamen, Spinal cord (cervical c-1), & Substantia nigra	1845
Colon	Sigmoid & Transverse	163
Esophagus	Gastroesophageal Junction, Mucosa, Muscularis	350
Heart	Atrial Appendage & Left Ventricle	321
Kidney	Cortex, Medulla	48
Liver	Liver	121
Lung	Lung	213
Muscle	Skeletal	280
Nerve	Tibial	217
Pancreas	Pancreas	38
Pituitary	Pituitary	207
Prostate	Prostate	80
Salivary Gland	Minor Salivary Gland	46
Skin	Not Sun Exposed (Suprapubic) & Sun Exposed (Lower leg)	639
Small Intestine	Terminal Ileum	10
Spleen	Spleen	13
Stomach	Stomach	43
Testis	Testis	144
Thyroid	Thyroid	228

Table S6

Table S6 (Related to Figure 5). RNA expression of the human GCGR from various tissues and specific cells
Total number of cells in each cluster used in figure 5B. Arranged according to number of cells

Liver tissues		Kidney tissues		**Pancreas tissues**	
**Cell type (cluster)**	**Number of cells**	**Cell type (cluster)**	**Number of cells**	**Cell type (cluster)**	**Number of cells**
Hepatocytes 1 (1)	1006	Tubule cells (1)	9620	Alpha cells (1)	309
a/b T-cells (2)	961	Tubule cells (2)	7329	Beta cells (1)	302
Hepatocytes 2 (3)	909	Tubule cells (3)	1974	Acinar cells (1)	272
Inflammatory Macs (4)	813	Proximal straight tubule cells (4)	1267	Alpha cells (2)	213
Hepatocytes 3 (5)	629	NK-T cells (5)	920	Ductal cells (1)	181
Hepatocytes 4 (6)	603	Glomerular parital epithelial cells (6)	734	Ductal cells (2)	175
Plasma cells (7)	511	Monocytes (7)	722	Alpha cells (3)	162
NK-like cells (8)	488	Distal tubule cells (8)	420	Alpha cells (4)	143
y/d T-cells 1 (9)	464	Collecting duct principle cells (9)	161	Beta cells (2)	142
Non-inflammatory Macs (10)	379	B cells (10)	144	Delta cells	135
Periportal LSECs (11)	327	Collecting intercalated cells (11)	77	Alpha cells (5)	82
Central Venous LSECs (12)	306			Acinar cells (2)	73
Portal endothelial cells (13)	211			Mesenchymal cells	68
Hepatocytes 5 (14)	202			Alpha cells (6)	57
Hepatocytes 6 (15)	152			Acinar cells (3)	54
Mature B-cells (16)	129			Alpha cells (7)	49
Cholangiocytes (17)	119			Ductal cells (3)	49
y/d T-cells 2 (18)	105				
Erythoid cells (19)	93				
Hepatic stellate cells (20)	37				

Table S6

Table S6: Key resources table showing reagents including software listed with identifiers.

REAGENT or RESOURCE	SOURCE	IDENTIFIER
Antibodies		
Glucagon Receptor Antibody LS-C403183	LSbio Seattle, Washington, USA	Cat# LS-C403183; RRID: AB_2890659
Anti-Glucagon Receptor (extracellular) antibody	Alomone Labs, Jerusalem, Israel	Cat# AGR-024; RRID: AB_2340975
Glucagon Receptor antibody	Biorbyt, Cambridge, UK	Cat# orb6093; RRID: AB_10920094
Rabbit Anti-Glucagon Receptor Polyclonal Antibody, Unconjugated	Bioss	Cat# bs-3945R; RRID: AB_10856630
Glucagon Receptor Polyclonal Antibody	Thermo Fisher Scientific	Cat# PA5-50668; RRID: AB_2636120
Rabbit Anti-Human Glucagon Receptor Polyclonal Antibody, Unconjugated	GeneTex Irvine, Californien, USA	Cat# GTX71693; RRID: AB_375890
Polyclonal Rabbit anti-Human GCGR / Glucagon Receptor Antibody	LSbio Seattle, Washington, USA	Cat# LS-C498919; RRID: AB_2890660
Anti-Mouse Glucagon receptor	TriChem (cedarlane), Skanderborg, Denmark	Cat# CL8893AP; RRID: AB_2890661
Glucagon Receptor (extracellular) Polyclonal Antibody	Thermo Fisher Scientific	Cat# PA5-77446; RRID: AB_2735850
Anti-GCGR polyclonal antibody	Atlas Antibodies, Bromma, Sweden	Cat# HPA071228; RRID: AB_2686368
Glucagon receptor antibody	Abcam, Cambridge, UK	Cat# ab75240; RRID: AB_1523687
Anti-Glucagon Receptor antibody	Abcam, Cambridge, UK	Cat# ab188743; RRID: AB_2890662
Myc-DYKDDDDK Tag monoclonal antibody	Invitrogen, Naerum, Denmark	Cat# MA1-91878; RRID: AB_1957945
horseradish peroxidase (HRP)-conjugated goat anti-rabbit	DAKO A/S, Glostrup, Denmark	Cat# P0448; RRID: AB_2617138
horseradish peroxidase (HRP)-conjugated rabbit anti-goat immunoglobulins	DAKO A/S, Glostrup, Denmark	Cat# P0449; RRID: AB_2617143
Donkey anti-Rabbit IgG (H+L), Alexa Fluor 546	Thermo Fisher Scientific, Slangerup, Denmark	Cat# A10040; RRID: AB_2534016
Alexa Fluor 488 conjugated goat anti-mouse IgG	Santa Cruz Biotechnology, Dallas Texas, USA	Cat# sc-514592; RRID: AB_2904166
Goat anti-Insulin	MyBioSource, San Diego, CA, USA	Cat# MBS448113; RRID: AB_2915923
Goat anti-somatostatin	Nordic Biosite, Copenhagen, Denmark	Cat# EB11971; RRID: AB_2915922
Mouse monoclonal IgG_1 κ anti-glucagon conjugated to Alexa Fluor® 488	Santa Cruz Biotechnology, Heidelberg, Germany	Cat# sc-514592; RRID: AB_2629431
Donkey anti-goat IgG conjugated to AlexaFluor 488,	ThermoFischer Scientific, Slangerup, Denmark	Cat# A11055; RRID: AB_2534102
Mab anti GAPDH	Merck KGaA, Darmstadt, Germany	Cat# G8795; RRID: AB_1078991
Biological samples		

Human liver tissue biopsies from individuals with non-alcoholic steatohepatitis	Fatty Liver Disease in Nordic Countries (FLINC)	https://clinicaltrials.gov/ct2/show/NCT04340817
Human renal tissue biopsies from healthy individuals	The European Nephrectomy Biobank (ENBiBA) project	https://www.era-online.org/en/diabesity/#toggle-id-5
Chemicals, peptides, and recombinant proteins		
X-tremeGENE9 Transfection Reagent	Roche Applied Science, Hvidovre, Denmark	Cat# 6365779001
4', 6-diamidino-2-phenylindole (DAPI)	Invitrogen, Naerum, Denmark	Cat# 10236276001
Epitope retrieval buffer	DAKO A/S, Glostrup, Denmark	
3,3'diaminobenzidine substrate	Vector Lab, Burlingame, Ca	Cat# SK-4100
cOmplete™, EDTA-free Protease Inhibitor Cocktail	Roche Applied Science, Hvidovre, Denmark	Cat# 4693132001
ECL™ Prime Western Blotting System	Amersham Biosciences, Amersham, UK	Cat# GERPN2232
Ketamine	MSD Animal Health, Madison, NJ, USA	Cat# 511485
Xylazine	Bayer Animal Health, Leverkusen, Germany	
^{125}I-Glucagon	Novo Nordisk, Bagsvaerd, Denmark	
Kodak NTB emulsion	VWR, Herlev, Denmark	Cat# KODK8895666
Kodak D-19 developer	VWR, Herlev, Denmark	Cat# KODK5158621
Dako Real™ Target Retrieval Solution	DAKO A/S, Glostrup, Denmark	Cat# S2031
Experimental models: Cell lines		
Human embryonic kidney (HEK) 293 cells	Constructs were established by Drs. Sanjay and Houghton [1]	N/A
Experimental models: Organisms/strains		
C57BL/6JRj mice	Janvier Laboratories, Saint-Berthevin Cedex, France	N/A
C57BL/6J^{Gcgrtm1Mjch} (Glucagon receptor knockout mice) and wild-type littermates	Gelling et al.,[2]	N/A
Recombinant DNA		
Full-length human GCGR, Myc-DDK-tagged cDNA	OriGene Technologies, Inc. Rockville, MD 20850, USA	Cat# RC211179
Mouse GCGR, Myc-DDK-tagged cDNA	OriGene Technologies, Inc. Rockville, MD 20850, USA	Cat# MR207767
Software and algorithms		
Axiovision program	Carl Zeiss, Oberkochen, Germany	https://www.micro-shop.zeiss.com/en/us/system/software+axiovision-axiovision+program-axiovision+software/10221/

MetaMorph software		https://www.moleculardevices.com/products/cellular-imaging-systems/acquisition-and-analysis-software/metamorph-microscopy
Zeiss Axioscope 2 plus	Brock & Michelsen, Birkerød, Denmark	https://www.zeiss.com/content/dam/Microscopy/us/download/pdf/end-of-service/upright/upright-axioskop-2-fs-plus-fs-mot-2.pdf
Other		
Sequence data, analyses, and resources related to cell-level gene expression analysis.		https://github.com/nicwin98/GCGR_Expression
Normalized hepatic single-cell RNA-sequencing data	MacParland et al.,[3]	https://github.com/BaderLab/HumanLiver
Raw renal scRNA-sequencing data	Liao et al.,[4]	https://github.com/lessonskit/Single-cell-RNA-sequencing-of-human-kidney

Supplementary References

1. Sanjay, A., et al., *Cbl associates with Pyk2 and Src to regulate Src kinase activity, αvβ3 integrin-mediated signaling, cell adhesion, and osteoclast motility.* 2001. **152**(1): p. 181-196.
2. Gelling, R.W., et al., *Pancreatic beta-cell overexpression of the glucagon receptor gene results in enhanced beta-cell function and mass.* Am J Physiol Endocrinol Metab, 2009. **297**(3): p. E695-707.
3. MacParland, S.A., et al., *Single cell RNA sequencing of human liver reveals distinct intrahepatic macrophage populations.* Nat Commun, 2018. **9**(1): p. 4383.
4. Liao, J., et al., *Single-cell RNA sequencing of human kidney.* Sci Data, 2020. **7**(1): p. 4.
